# The Role of Biomarkers in Atherothrombotic Stroke—A Systematic Review

**DOI:** 10.3390/ijms22169032

**Published:** 2021-08-21

**Authors:** Sebastian Andone, Zoltan Bajko, Anca Motataianu, Oana Mosora, Rodica Balasa

**Affiliations:** 1 Doctoral School, ‘George Emil Palade’ University of Medicine, Pharmacy, Science, and Technology of Targu Mures, 540142 Targu Mures, Romania; sebastian.andone@umfst.ro (S.A.); rodica.balasa@umfst.ro (R.B.); 2 1st Neurology Clinic, Mures County Clinical Emergency Hospital, 540136 Targu Mures, Romania; anca.motataianu@umfst.ro (A.M.); oanamosora.92@yahoo.com (O.M.); 3 Department of Neurology, University of Medicine, Pharmacy, Science and Technology Targu Mures, 540136 Targu Mures, Romania

**Keywords:** stroke, biomarkers, atherothrombotic, atherosclerosis

## Abstract

Stroke represents the primary debilitating disease in adults and is the second-highest cause of death worldwide. Atherosclerosis, the most prevalent etiology for vascular conditions, is a continuous process that gradually creates and develops endothelial lesions known as atherosclerotic plaques. These lesions lead to the appearance of atherothrombotic stroke. In the last decades, the role of biological biomarkers has emerged as either diagnostic, prognostic, or therapeutic targets. This article aims to create a list of potential biomarkers related to atherothrombotic stroke by reviewing the currently available literature. We identified 23 biomarkers and assessed their roles as risk factors, detection markers, prognostic predictors, and therapeutic targets. The central aspect of these biomarkers is related to risk stratification, especially for patients who have not yet suffered a stroke. Other valuable data are focused on the predictive capabilities for stroke patients regarding short-term and long-term prognosis, including their influence over the acute phase treatment, such as rt-PA thrombolysis. Although the role of biomarkers is anticipated to be of extreme value in the future, they cannot yet compete with traditional stroke neuroimaging markers but could be used as additional tools for etiological diagnosis.

## 1. Introduction

Over the last few years, the role of biomarkers, or biological markers, has significantly grown as new molecules have emerged as potent candidates.

The first description of a biomarker established by expert consensus (Committee on Biological Markers of the National Research Council) appeared in 1987 and stated, “Biological markers are indicators signaling events in biological systems or samples” [1].

As the biomarker domain evolved progressively, so did the need for a complete definition.

The National Institutes of Health Biomarkers Definitions Working Group proposed a new definition in 2001: “A characteristic that is objectively measured and evaluated as an indicator of normal biological processes, pathogenic processes, or pharmacologic responses to a therapeutic intervention” [2].

An essential role of biomarkers is integrated into the notion of precision medicine—this grants breakthrough opportunities for the advancement of research and technology used in healthcare systems.

The concept of precision medicine is based on the adjustment of medical therapy to a specific subgroup of patients according to specific conditions. These conditions can involve the use of biomarkers that are specific to a particular dysfunction.

The increased speed of discovering new biomarkers will enable the role precision medicine has in clinical practice [3] to increase over time.

Stroke is the second leading cause of death worldwide and is the primary debilitating disease that affects adults, with up to 50% of patients being chronically disabled [4].

A stroke is defined as a group of symptoms or signs that correspond to the focal loss of cerebral function, with no other apparent cause.

However, the term stroke is so vaguely defined that it required specific definitions for each type (ischemic or hemorrhagic) and each subtype (silent, infarction) [5].

Our main study focus, the atherothrombotic stroke, appears when a thrombus occludes a large intracranial or extracranial vessel, usually at the level of an atherosclerotic plaque or an endothelial injury.

Atherosclerosis, a chronic inflammatory disease of the blood vessels, is the most prevalent etiology for vascular conditions such as ischemic heart disease and stroke.

Atherosclerosis is defined by the gradually advancing dysfunction of the large arteries creating specific endothelial lesions, the atherosclerotic plaques.

Atherosclerotic plaques were initially thought to be just lipid deposits in the intima or the arterial wall. However, this concept was proven to be flawed, as more evidence appeared that linked the atherosclerotic process with inflammation.

The entire process is much more complex, as more and more markers involved in this process are found, proving that atherosclerosis is much more of a complicated matrix of mechanisms than ever before.

The initial phase of atherosclerosis is represented mainly by lipids deposits, with no real hemodynamic impact. However, the leading promoter of this phase is represented by endothelial dysfunction, which initiates the atherosclerotic process and contributes to the development of the subsequent phases.

The endothelial dysfunction is usually an injury of the arterial wall created by either physical (shear stress, trauma) or chemical factors (elevated LDL, production of reactive oxygen species).

The lipids deposits start to appear inside the arterial intima, promoted by increased endothelial permeability caused by the initial lesion. Following this, proinflammatory cytokines are released as a response to the LDL particles. These cytokines increase the secretion of several pro-atherothrombotic molecules, such as adhesion molecules.

Drawn by this, inflammatory cells infiltrate the vessel wall, mostly monocytes that differentiate into macrophages, but also neutrophils and T cells, to a lesser degree. The macrophages engulf the already present LDL, transforming into foam cells, which continue the cycle of cytokine release, further intensifying the atherosclerotic plaque development.

The plaque evolution represents the second phase. All the previously mentioned effects from the initial phase continue to take place. In contrast to the first phase, the second one is beginning to impact hemodynamic parameters directly.

**Figure 1:**
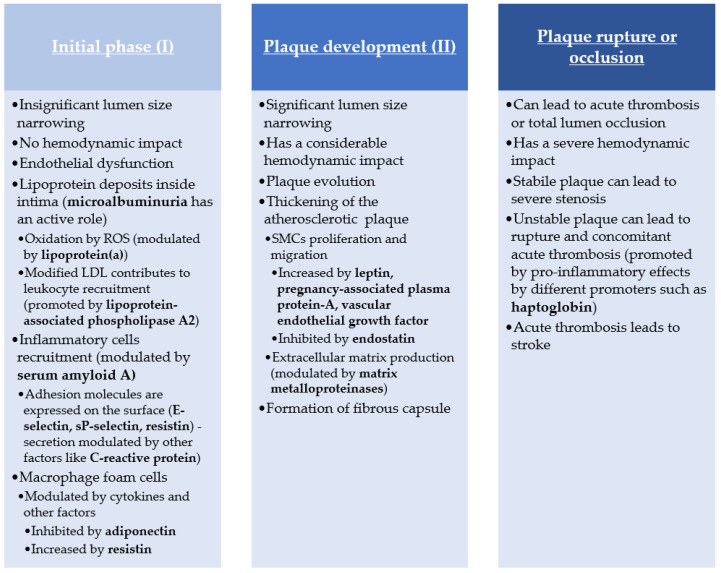
Atherosclerosis pathogenesis pathway.

This process is caused by the thickening of the atherosclerotic plaque produced by the migration and proliferation of smooth muscle cells and the formation of the extracellular matrix. Gradually, the atherosclerotic plaques change from a primarily fatty structure to a fibrous structure. This process can develop over several years or decades to such an extent that it could lead to severe arterial stenosis. The final phase of the atherosclerotic plaques consists of plaque rupture or complete lumen occlusion by the plaque. In time, due to the hemodynamic stress and continuous metabolic changes that happen inside the plaque, the plaque becomes unstable, up to the moment when the fibrous cap ruptures. This final event most often leads to the formation of an acute thrombus [6,7,8,9] (Figure 1).

The hyperacute phase of stroke is initiated by this process, resulting in sodium accumulating inside the neurons because of reduced ATPase activity. This is responsible for the cell swelling that defines cytotoxic edema. Calcium levels also increase inside the cell, accelerating cell death caused by glutamate and dopamine toxicity. If reperfusion happens during the hyperacute phase, it can save the penumbra tissue that is still viable. However, this can also lead to increased osmotic solutions passing through the BBB because of increased permeability, which can lead to increased brain edema.

After the first 6 h, the acute phase of the stroke begins and lasts for the next 3–4 days. This stage focuses mainly on the brain tissue surrounding the infarct zone, which is usually caused by different processes than the hyperacute phase. Now the inflammatory response is the agent responsible for secondary injury as the brain lesions advance. The neuroinflammation is promoted by the cell detritus following cell death and also by elevated ROS that activates microglia and astrocytes. This disrupts even more BBB and increases the permeability. Activated inflammatory cells release cytokines that create a perpetual cycle of inflammation.

After approximately seven days from the onset of the stroke, the next stroke phase begins; the subacute phase. This stage is dependent on neuroinflammation, but instead of proinflammatory activity, it changes the reaction to anti-inflammatory. This stabilizes the BBB and initiates the neuroregenerative response. One of these mechanisms of action is angiogenesis that helps create new blood vessels, an effect modulated by a couple of factors like hypoxia or specific molecules.

**Figure 2:**
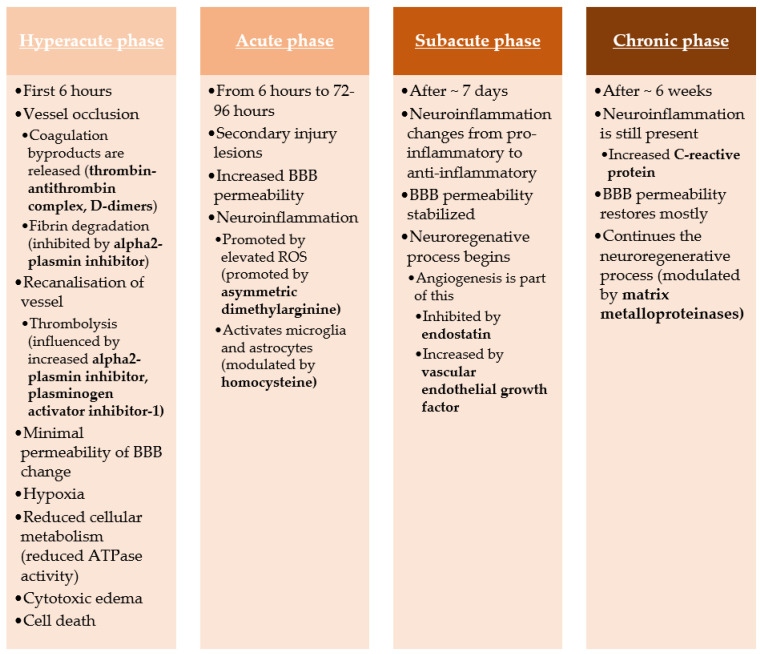
Stroke pathogenesis pathway.

The final phase of stroke is the chronic stage that sets after approximately six weeks from the initial event. The BBB permeability is restored to almost the same level as before, even if the neuroinflammation response is still present, as cytokines levels are still raised above normal [8,9,10,11] (Figure 2).

The purpose of this article is to review the current biomarkers related to atherothrombotic stroke, focusing on their roles as a risk factor, detection marker, and prognostic predictor, and therapeutic targets.

## 2. Materials and Methods

We undertook a systematic review following PRISMA (Preferred Reporting Items for Systematic Reviews and Meta-analyses) guidelines (http://www.prisma-statement.org/, accessed on 1 June 2021).

The search strategy included two journal databases, NCBI PubMed and Google Scholar.

We identified the following 23 biomarkers that are strongly related to atherothrombotic stroke: C-reactive protein, thrombin-antithrombin complex, alpha2-plasmin inhibitor, plasminogen activator inhibitor-1, soluble P-selectin, E-selectin, adiponectin, leptin, resistin, D-dimers, homocysteine, asymmetric dimethylarginine, lipoprotein (a), lipoprotein lipase, lipoprotein-associated phospholipase A2, haptoglobin, serum amyloid A, microalbuminuria, pregnancy-associated plasma protein-A, matrix metalloproteinases (MMP-2, MMP-9), endostatin, and vascular endothelial growth factor.

Following that, we performed a query using the search terms: (ischemic stroke + variable biomarker name) OR (atherothrombotic stroke + variable biomarker name) OR (atherosclerosis + variable biomarker name) OR (stroke treatment + variable biomarker name).

The automation process was performed using a script that can fetch information based on several keywords. Using it, we compiled lists of possible articles for each keyword. The lists were truncated together, and the duplicate items were automatically deleted.

In addition, we used both the full variable name, the alternative names, and the common abbreviations as search variables in separate search queries to reduce the limitations derived from the search engines.

No data or language restrictions were applied to the search engines. We included studies on humans, experimental studies on animals, in vitro studies, and in silico studies. Duplicate findings were removed by manual revision or by using automation tools.

**Inclusion criteria**: we included all studies that provided statistical analysis (descriptive statistics, comparison tests, categorical data tests, linear regression, uni- and multivariate statistics) between groups of patients or animals (in the case of experimental in vivo studies) with ischemic stroke and a control group (either healthy subjects or subjects that had a different therapeutic approach compared to the previous group) regarding a biomarker as measured in venous blood.

For in vitro and in silico studies, we included studies that had sufficient information to summarize a conclusion regarding the analyzed effect of the biomarker on different processes that are directly connected with ischemic stroke.

**Exclusion criteria**: we excluded all the studies that provided analysis regarding only one subtype of stroke, other than the atherothrombotic subtype (e.g., the effects of a specific biomarker analyzed only in the cardioembolic or small-vessel disease subtypes, without any reference or comparison to atherothrombotic subtype). In addition, hemorrhagic stroke studies were excluded if they offered no parallel to an ischemic stroke group of patients from the same study cohort.

**Figure 3:**
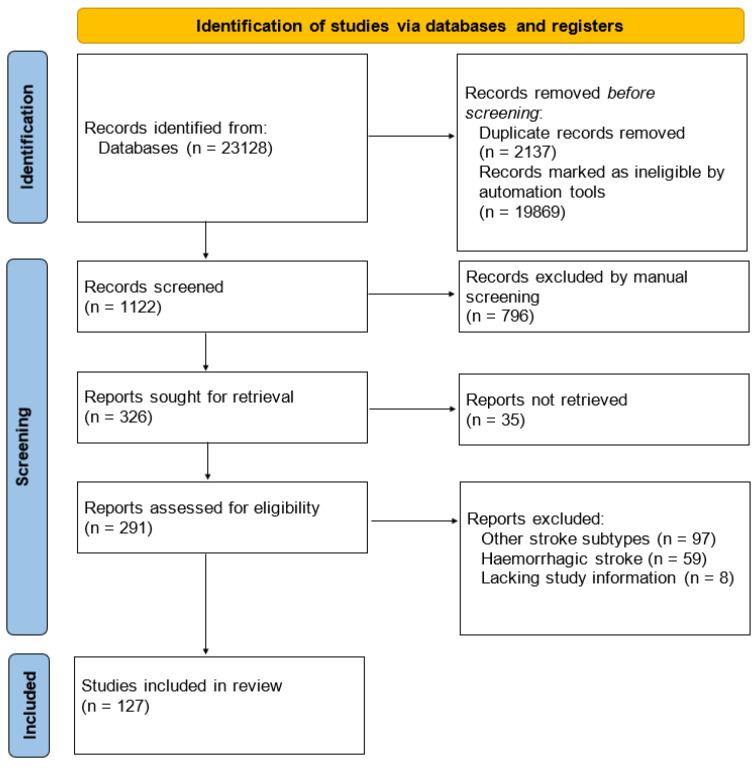
PRISMA flow diagram for the systematic review.

After screening, duplicate removals, and exclusions, we identified 127 studies that we included in our review (Figure 3).

## 3. Discussion

We discuss each biomarker, assessing its role and its function as a biomarker of stroke.

Regardless of the pathology, a biomarker has some universal characteristics [12] it should comply with (Table 1).

**Figure 4:**
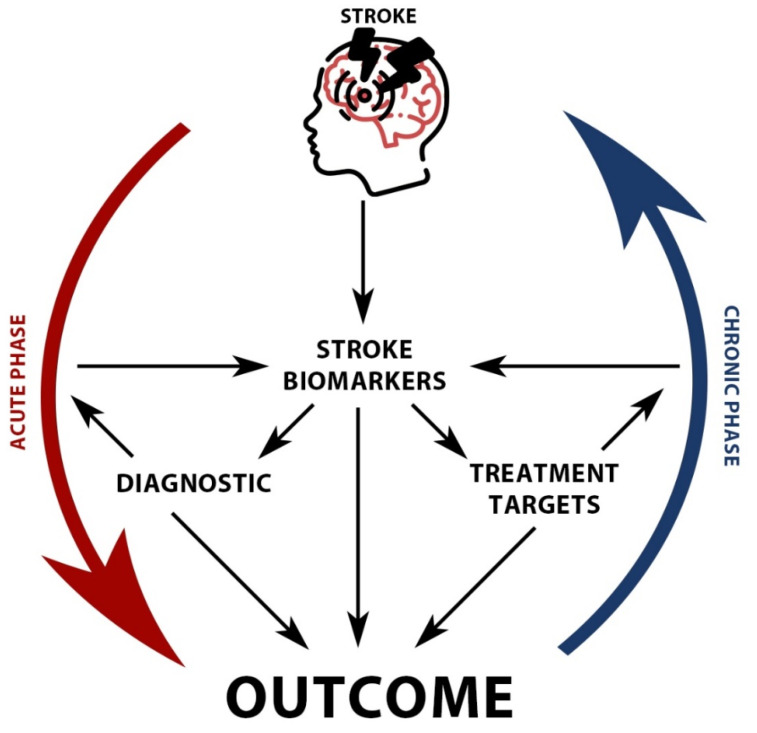
Stroke biomarkers roles and interrelationships.

Using these, we analyzed each biomarker’s role as a risk factor in the pathogenesis of the disease, the molecular response at the onset of the disease regarding that specific marker, the predictive prognostic factor, and if that biomarker affects or is affected by the stroke therapy (Figure 4).

**C-reactive protein (CRP)** represents an acute-phase biomarker of inflammation. The liver mainly secretes this marker in the acute phases of inflammation, but the local site of the injury also produces it in lower levels [13]. In addition, high-sensitivity CRP (hs-CRP) can be used to identify a low-level inflammatory response [14].

Evidence suggests that CRP modulates adhesion molecules and nitric oxide in vessel wall cells, provoking a self-maintained atherothrombotic process. Also, CRP induces the expression of PAI-1 in the vascular muscle cells and seems to influence intraplaque angiogenesis, increasing the risk of intraplaque hemorrhage [15].

Several studies have shown the association between high CRP values and the increased risk of developing an ischemic stroke [16,17].

In the elderly, CRP can act as an independent predictor of a future stroke or transient ischemic attack (TIA) [18].

Apart from the risk of stroke, high CRP values are predictive of cognitive impairment [19]. Even more so, increased CRP alongside increased homocysteine was associated with the risk of developing post-stroke depression after one year from the stroke [20].

High CRP measured in the interval between 12 and 24 h from the onset correlates with poor outcome and increased incidence of other cerebrovascular or cardiovascular events [21].

Admission CRP levels were associated with poor functional outcomes at discharge as measured by NIHSS score as an independent predictor [22]. Furthermore, discharge CRP levels are also an independent biomarker for poor prognostic [23].

Like other proinflammatory markers, CRP is associated with poor short-term functional outcomes [24], poor long-term outcomes, and increased 3-month mortality [15,25].

Besides age and NIHSS severity, increased CRP values are an independent marker for mortality [25], especially in elderly patients, although it is non-specific [26].

As for treatment, statins have been proven to reduce hs-CRP values, independently of their effects on lipids [27].

Further studies are required to detect potential therapeutic candidates for reducing CRP levels in stroke, given its prognostic predictor value.

**Thrombin-antithrombin complex (TAT)** is a protein association between thrombin and antithrombin formed in the case of a coagulation event. The complex appears when the antithrombin is combined with the newly produced thrombin in a 1:1 ratio, creating the TAT biomarker. During this process, the enzymatic activity of thrombin ceases to exist without the possibility of reversal of the mechanism.

Although the relationship between TAT and stroke is uncertain, as TAT is more of a byproduct of coagulation, increased thrombin causes brain edema and the disruption of BBB with the migration of inflammatory cells [28].

Naifang et al. analyzed the performance of this biomarker in acute ischemic stroke alongside d-dimer assay and concluded that high TAT values represent an independent poor prognostic factor.

The median values of TAT and D-dimer were significantly higher in stroke patients compared to healthy subjects. In addition, high plasma TAT levels alongside d-dimer were correlated to increased NIHSS scores. The TAT values were significantly higher in patients with poor outcomes compared to patients with good outcomes [29].

Fernandez-Cadenas et al. evaluated TAT in patients treated with recombinant tissue plasminogen activator (rt-PA) and observed that lower values of TAT had been correlated with improved recanalization rates regardless of the time of rt-PA administration [30].

**Alpha2-plasmin inhibitor (a2-PI)** represents a protease inhibitor that belongs to the serpins, a superfamily of similar structure proteins, and it is the prime inhibitor of plasmin [31]. Alongside other coagulation factors, it is produced in the liver and can be affected by hepatic disorders, such as cirrhosis, leading to increased hemorrhagic risk. a2-PI is an effective plasmin inhibitor that increases the resistance of fibrin degradation. [32].

High plasma a2-PI values are found in acute atherothrombotic stroke patients but are also associated with an increased risk of stroke recurrence [33].

Increased levels of a2-PI have also been associated with a lower rate of rt-PA reperfusion [34].

In an experimental study, Nagai et al. observed that by administering human plasmin to mice with MCA occlusion-induced stroke, the focal ischemic brain lesion volume was significantly reduced [35].

**Plasminogen activator inhibitor-1 (PAI-1)**, known as **endothelial plasminogen activator inhibitor**, is another serine protease inhibitor that functions to inhibit plasminogen activators as tissue plasminogen inhibitor or urokinase.

PAI-1 appears as an acute phase reagent in stroke, secondary to inflammation, leading to brain lesions progression. Also, elevated PAI-1 causes thrombolytics resistance, especially in platelet-rich thrombosis. PAI-1 can be linked to hypercoagulability as a possible cause of ischemic stroke [36].

Increased PAI-1 levels have been associated with obesity, which is a stroke risk factor. Additionally, because of the connection between stroke and obesity, PAI-1 has also been strongly linked with both first-time stroke risk and stroke recurrence risk [37].

The study of genetic polymorphism of PAI-1 and stroke showed that PAI-1 4G/5G polymorphism was associated with increased stroke risk in the adult population [36]. Another study tried to find any difference between good and poor outcome groups of stroke patients treated with rt-PA but found no statistically significant difference regarding PAI-1 polymorphism [38]. However, another study showed that the PAI-1 5G/5G genotype is associated with an increased risk of hemorrhage after rt-PA treatment. Furthermore, PAI-1 levels were increased significantly in patients who had ASPECTS scores below 7 points [39].

In an experimental study on hypertensive rats subjected to MCA occlusion, it was observed that by administering a PAI-1 inhibitor, there was a reduction of brain injury [40].

**Soluble P-selectin (sP-selectin)** or **CD62P** is an endothelial cell receptor expressed on the surface with a role in molecule adhesion and can also be found on platelets [41,42].

sP-selectin elevated plasma values appear shortly after platelet aggregation, indicating thrombotic dysfunctions, showing the extent of platelet activation. Not only are platelets mediated by sP-selectin, but inflammatory cells such as neutrophils are also subject to its cell adhesion effect, thus influencing the reperfusion lesion [43].

It has been stated, and also observed in experimental studies, that sP-selectin has a critical role in the initiation of atherosclerosis, especially in cell tethering and leukocyte rolling [41].

Increased values of sP-selectin were also found in patients with aortic atherosclerosis [43].

Compared to the normal control group, stroke patients had significantly higher levels of sP-selectin [44]. Moreover, raised levels of sP-selectin were found in patients with progressive ischemic stroke [43].

Increased values of sP-selectin were found in the acute phase of ischemic stroke, with a decrease on a three month follow-up [43].

Pawelczyk et al. studied the effect of statin therapy on platelet activity biomarkers and saw a significant decrease in sP-selectin values in patients with a hyperlipidemic stroke profile [45].

**E-selectin** or **endothelial-leukocyte adhesion molecule 1 (ELAM-1)** or **CD62E** is a selectin cell adhesion molecule expressed on endothelial cells that have been previously activated by cytokines [46]. Even though it is similar to other selectin molecules in that it plays a significant role in inflammation, especially in blood leukocyte recruitment and adhesion, E-Selectin is different from sP-selectin because of its much slower mechanism of action. While P-selectin is expressed on the cell surface in minutes, E-selectin is produced on demand, and it takes up to 2 h until expressed on the surface.

E-selectin also increases inflammatory cells’ adhesion to vessel walls in areas that are receptive to the atherosclerotic process and could lead to the evolution of plaques [47].

However, the production of E-selectin seems to be activated by the sP-selectin under stimulation from tumor necrosis factor-alpha and interleukin-1 [48,49].

The genetic polymorphism of E-selectin plays an important role, as E-selectin S128R and L554F genotypes are associated with susceptibility to ischemic stroke. In addition, the association of minor alleles increases the risk for ischemic stroke even more [50].

Huang et al. published an experimental study on mice subjected to transient intraluminal occlusion of the middle cerebral artery, in which he injected anti-E-selectin monoclonal antibodies. He showed that by blocking the E-selectin, the mortality, infarct volumes, and cerebral neutrophil accumulation were significantly reduced [47].

In human subjects, E-selectin was proven to be an independent predictor of poor outcomes in patients who had a stroke by analyzing the modified Rankin Scale after three months from the event [51].

**Adiponectin (APN)**, a member of the family of adipokines, is involved in the regulatory mechanisms of glucose and fatty acid metabolism pathways. It is primarily produced in adipose tissue and, in smaller quantities, in muscle and brain tissue. APN has a dual role: it acts as a hormone and also as a cytokine. The hormonal part involves regulating glucose and lipid metabolism, while the cytokine role involves anti-atherogenic and anti-thrombotic characteristics [52].

The molecular structure of APN is similar to complement protein C1Q, having three circulating forms with different molecular weights. APN also inhibits the adhesion of monocytes and can suppress the formation of macrophage foam cells, in strong connection with the atherosclerotic process [53].

In a meta-analysis regarding APN, Gorgui et al. concurred that higher APN values were associated with a lower prevalence of carotid plaques [53].

In another study, APN levels were measured between two groups: stroke patients and the control group. Stroke patients showed a lower level at admission compared to the healthy ones [54]. However, acute ischemic stroke patients that had a higher value of APN on admission were more likely to have a poor outcome and increased risk of mortality [55].

Efstathiou et al. studied the association between APN and five-year survival prognosis in a group of first-ever ischemic stroke patients and showed that a low level of APN is associated with increased mortality [56].

Although APN seems to be a protective biomarker as far as it concerns the risk of stroke and the atherosclerosis process, no APN agonist has been developed yet to study the benefic effects it could have.

**Leptin**, the first adipokine discovered (1994), is mainly produced by adipocytes and enterocytes and has a role in energetic regulation and hunger inhibition. To achieve its function, leptin activates cell receptors located in the hypothalamus nuclei and, as a result, is being able to mediate the feeding cycle. Similar to APN, it has two roles: hormone and cytokine [57].

Its role in the atherosclerotic process is well defined, primarily by endothelial dysfunction, increasing platelet aggregation, and affecting the smooth vascular cells, leading to hypertrophy and proliferation. It has a stimulator role on CD4+ and CD8+ T cells, increasing other cytokines and CRP proinflammatory [55].

In experimental studies with mice, leptin was proven to have a modulatory role in activating T cells. The same study also showed that mice with leptin receptor deletion had a reduction of infarct inflammation and the size of atherosclerotic plaques [58]. In stroke patients, both ischemic and hemorrhagic types, the value of leptin was significantly higher compared to healthy subjects [54]. Another prospective study on patients with a history of stroke showed that higher leptin values were associated with higher stroke incidence [59].

Leptin levels measured at discharge were associated with increased incidence of post-stroke depression in the following month [60].

Leptin to APN ratio has been considered a possible marker for predicting common carotid artery intima-media thickness [52]. In addition to this, an increased leptin/adiponectin ratio at admission is associated with good outcomes in atherothrombotic stroke patients [61].

Lifestyle changes and several drug classes, such as statins and oral antidiabetic drugs, can lower leptin values [62].

**Resistin** or **adipose tissue-specific secretory factor (ADSF)** is another member of the adipokine family, produced in adipose tissue in rodents, while in primates and humans, it is mostly produced by the monocytes and macrophages. Its role is slightly different in each species, but it mainly causes insulin resistance [63,64].

Resistin has a pro-atherosclerotic function by modulating endothelial cells by decreasing nitric oxide while increasing the adhesion molecules expression. Additionally, resistin also increases lipid accumulation in macrophages, thus playing an essential role in initiating the atherosclerotic process [65].

The relationship between resistin and atherothrombotic-related diseases such as cardiovascular and coronary heart disease has been clearly established in individuals, regardless of ethnicity and race [66].

However, a couple of studies have focused on specific groups, such as the Japanese, where high resistin values were found to be an independent risk factor for atherothrombotic stroke in the general population. The presence of diabetes or hypertension additionally increased the risk of ischemic stroke [64].

Furthermore, genetic polymorphism of resistin adds an extra layer of complexity to this connection, as resistin genotype −420 (C > G) is a supplementary stroke risk factor in type 2 diabetic Japanese patients [67].

Gender and age differences also exist, as in postmenopausal women, where high resistin levels are associated with an increased risk of ischemic stroke [65].

In women patients that had already developed an ischemic stroke, a higher resistin value was positively correlated with stroke severity using NIHSS score [68].

In the early phase of an acute ischemic stroke, high resistin values were found, indifferent of age or sex [69]. Another study confirmed the same observation, which additionally showed a further ethnic difference in stroke patients, with a significant association between resistin values and acute ischemic stroke in the Asian population, but not in the Caucasian population [70].

In atherothrombotic stroke patients, increased resistin values were associated with increased disability and increased five-year mortality risk as an independent predictive factor [71].

As a treatment option, resistin experimental studies on mice were performed, showing that resistin peak values are around 12 h from the stroke onset, reversing to basal values at 24 h. By administering a dose of resistin to the mice, they significantly reduced brain edema by suppressing apoptosis and oxidative stress [63].

This data suggests that future studies should be performed on humans to confirm the same findings, which could be of clinical importance.

**D-dimers (DDs)** represent a fibrin degradation product that appears in the blood after a clot is affected by fibrinolysis. The small protein is formed by combining two D fibrin fragments using a cross-link [72].

DDs are byproducts of secondary fibrinolysis with a significant clinical role, especially in deep vein thrombosis and pulmonary embolism. In stroke, DDs are mainly found to increase in the cardioembolic subtype, although they are not specific and can be found in other subtypes. In atherothrombotic stroke, DDs can be elevated, especially if the thrombus has a high platelet count [73].

A review by Ohara et al. showed that DDs have a unique role in the etiological diagnosis of stroke, especially in active, occult cancer and undetected atrial fibrillation [74].

Alongside CRP and soluble receptor 1 of tumor necrosis factor alfa (sTNFR-1), DDs are independent factors for poor functional prognosis at three months [73].

A review including over 22,000 patients concluded that a higher DDs value is more likely to be associated with an increased risk of ischemic stroke [75].

A study compared a group of patients with progressing stroke with a group of patients with no progression of symptoms and showed a significant difference in the mean value of DDs in the progressive stroke group, having a two-fold increased value [76].

However, DDs cannot be solely used for the diagnosis or prognosis prediction of stroke, as it is neither specific nor sensitive enough because of the limited evidence [77].

In patients who received thrombolytic treatment, individuals with poor outcomes after rt-PA had higher D-dimers values than patients with a good outcome, suggesting that d-dimer can have an acute predictive value in this specific category of patients [78].

**Homocysteine (Hcy)** is an alpha-amino acid, which is generated during methionine metabolism and is used for the biosynthesis of cysteine using the B6 vitamin (pyridoxine) as a reaction co-factor [79]. Among other pathogenic roles, homocysteine has a role in atherogenesis and endothelial cell injury.

Hcy seems to affect microglia proliferation and the capacity of neurons to transmit signals; however, the mechanism of action is not yet understood [80].

Hyperhomocysteinemia has been correlated with cardiovascular disease, including heart attacks and stroke [81]. The increased level of Hcy can be determined by a deficit in dietary intake of vitamin B12 and folic acid [82].

In a study published by Harris et al., Hcy seems to be a risk factor for increased stroke severity, as higher blood values were correlated with higher NIHSS scores. The same study stated that they found a 14.4 times higher risk for patients with high Hcy levels to have an NIHSS score >5 [83]. 

In a meta-analysis regarding the Chinese population, there was also evidence that higher serum Hcy levels are a risk factor for ischemic stroke [84]. Huang et al. showed that in the Chinese adult population, there was a significant reduction of first stroke risk in individuals that had hypertension when lowering the total Hcy percentage levels. In order to reduce the total Hcy, they used a double-blind, randomized, controlled trial and assigned one group hypertensive drugs combined with folic acid, while the control group was given hypertensive drugs with no folic acid. Neither group received B vitamin supplements [80].

Serum Hcy levels measured at admission were also associated with depression three months after the stroke event [85].

Total Hcy levels were correlated with the subgroup of patients with extensive artery atherosclerosis. In this group, there was also an increase in the risk of recurrence of stroke in patients that had high total Hcy levels during the convalescent phase of the stroke [86]. Furthermore, stroke recurrence risk and increased mortality were associated with elevated plasma Hcy levels [87].

**Asymmetric dimethylarginine (ADMA)** is a derivate product from the normal protein metabolism, being an endogenous inhibitor of nitric oxide (NO) synthesis in the vascular endothelium [88].

ADMA usually increases after acute stroke and is associated with and contributes to brain infarction development by reducing cerebral perfusion and inducing inflammation and oxidative stress [89].

Several studies have shown that increased ADMA levels are associated with the atherosclerosis process and endothelial dysfunction [88,90].

It has been pointed out that ADMA could be a possible risk biomarker for ischemic stroke, as healthy subjects had significantly lower ADMA compared to ischemic stroke patients [91]. Also, higher values of ADMA and decreased levels of NO could be used as independent risk factors for ischemic stroke [92]. In addition, another study showed that increased ADMA values are more likely to be associated with increased risk in young patients [93].

In a study carried out using middle-aged Japanese men, ADMA has proven to be significantly associated with increased predicted stroke risk, showing its usefulness as a possible screening marker in this specific population [94]. In addition, Worthmann et al. studied the effects of thrombolysis with rtPA over ADMA levels and showed that patients who received rt-PA had lower ADMA values than the placebo-treated group. However, further studies are required to prove whether ADMA has a role in the thrombolysis success rate [95].

Higher values of both ADMA and its analog, symmetric dimethylarginine (SDMA) in the first 72 h within stroke onset have been associated with a poor outcome 90 days after the event [89].

In order to reduce its possible effect on stroke risk, it is possible to decrease ADMA in blood levels by administering folic acid and B12 vitamins to patients [96].

**Lipoprotein (a) (Lp(a))** is a variant of LDL (low-density lipoprotein) that contains apolipoprotein (a) linked with apolipoprotein (B) on the surface of the particle [97].

The pathogenic mechanism of action of Lp (a) in stroke has not been completely identified, but there is an apparent pro-atherothrombotic and antifibrinolytic effect. Some of this influence can be explained by the proinflammatory impact of oxidized phospholipids, although further studies are required [98].

Multiple studies have identified Lp (a) as a risk factor for atherosclerosis, stroke, and other related atherothrombotic conditions like coronary heart disease [99,100]. In addition, case-control studies showed that higher Lp (a) values were found in stroke patients than in healthy controls [99].

A meta-analysis containing over 90,000 patients showed that Lp (a) is a consistent risk factor for ischemic stroke, and it is of high relevance, particularly in young patients [101].

Another large study involving over 49,000 patients included in the Copenhagen General Population Study and more than 10,000 patients from the Copenhagen City Heart Study concluded that high levels of Lp (a) and LPA genotypes are associated with increased risk of ischemic stroke, but at a lower rate compared to ischemic heart disease [102].

As a prognostic biomarker, there seems to be an association between high levels of Lp (a) and poor short-term outcomes in stroke patients. However, this statement needs to be further verified by future studies [98].

Lange et al. included 250 patients with an NIHSS range between 1 and 4 and showed that individuals with elevated Lp (a) levels are associated with a higher risk for a recurrent vascular event, especially in patients with first-time ischemic stroke [103].

Even though expert opinions are mixed, a recent meta-analysis of over six randomized trials proved that statin treatment significantly increases Lp (a) values in the blood [104].

**Lipoprotein lipase (LPL)** is a water-soluble enzyme genetically similar to other lipases like pancreatic or hepatic lipase, and its primary function is to hydrolyze triglycerides in lipoproteins. The LPL gene is found on chromosome 8 (8p22) and is expressed in muscle cells, adipocytes, and myocytes. Although the relationship between LPL and atherosclerosis is not fully understood, there is evidence that suggests that LPL has proatherogenic effects. Variations of the LPL gene can alter its enzymatic properties and activity role, especially as a stroke risk factor. Several common variations have been found and studied [105].

The presence of the HindIII polymorphism variant in the Asian population showed a decreased ischemic stroke risk, while the non-Asian population showed an increase in ischemic stroke risk [106].

Similarly, rs320 and rs285 variants proved to lower the risk of stroke more than other genetic variants found in the general population [107].

Another mutation with a protective role is Ser447Stop, which not only reduces the risk of ischemic stroke but also has an effect on atherosclerosis process development. The same mutation also seems to be a genetic biomarker specific for atherothrombotic stroke [108].

**Lipoprotein-associated phospholipase A2 (Lp-PLA2)** is a calcium-dependent lipase that travels linked to LDL and, in a smaller proportion, with HDL. It is also known as platelet-activating factor acetylhydrolase (PAF-AH). Its primary role is to hydrolyze oxidized phospholipids, and by doing so, creates byproducts involved in plaque inflammation and endothelial dysfunction leading to the atherosclerotic process [109].

Increased activity of Lp-PLA2 was found in patients who developed a transient ischemic attack (TIA) because of large-artery atherosclerosis. Furthermore, both large-artery disease and high levels of Lp-PLA2 are associated with increased risk for stroke in TIA patients [110].

Elevated Lp-PLA2 can also predict the risk for developing intracranial large-vessel occlusions in patients that have already had a TIA or are known to have intracranial arterial stenosis. [111]. After a first-time stroke, the risk of recurrent stroke after TIA is strongly associated with increased Lp-PLA2 levels [112]. This continuous connection between the recurrence risk and Lp-PLA2 makes it a useful probable biomarker for future clinical studies.

Several different studies have concluded that increased Lp-PLA2 is associated with both atherosclerotic stroke risk and other atherosclerotic disorders, such as coronary heart disease [109,113].

Racial differences have also been studied, showing that Lp-PLA2 high values are a risk factor for ischemic stroke in the Han Chinese population for both occurrence and recurrence [114]. Another study proved an association between elevated Lp-PLA2 and the risk of atherosclerotic stroke in non-Hispanic white people compared to other race-ethnic groups [115].

Increased Lp-PLA2 values are further associated with modest long-term prognostic, especially with mortality regardless of cause within one year after stroke [116].

**Haptoglobin (Hp)** is an acute-phase plasma protein that forms a complex by adhering to free hemoglobin (Hb) released by damaged erythrocytes, with the role of inhibiting the harmful impact, especially its oxidative damage related to internal organs accumulation of Hb. This complex is later removed from the circulation, usually in the spleen [117]. Hp has a genetic polymorphism with three different isoform proteins.

Of these, Hp2-2 has a higher Hb binding affinity than Hp1-1 and has an increased proinflammatory effect by increasing IL-10 secretion, leading to the destabilization of plaque during atherosclerosis development [118].

The Hp2 allele has been associated with increased cardiovascular risk between these genotypes, especially with premature deaths after first-time ischemic stroke [119]. The same allele has also been associated with the presence of unstable carotid atherosclerotic plaques with high susceptibility for ischemic stroke and transient ischemic attacks [120].

Regardless of genotypes, Hp levels over 1040 ug/mL in the first 12 h were strongly associated with atherothrombotic stroke with extremely high sensitivity and specificity [118].

More specifically, the Hp 2-2 genotype was strongly associated with carotid artery stenosis found using color doppler ultrasound in patients with ischemic stroke, compared to other genotypes such as Hp1-1, proving its usefulness as a potential genetic biomarker [121].

Additionally, in a study regarding Hp as a therapeutic target, patients that were found to have the Hp 2-2 genotype received vitamin E and showed a decrease in cardiovascular events, with a rapid increase of incidence when the treatment was stopped [122].

**Serum amyloid A (SAA)** is an acute-phase protein and is part of the family of apolipoproteins bound with high-density lipoprotein (HDL). SAA has different functions varying from the recruitment of inflammatory cells to cholesterol transportation. A known ischemic stroke risk factor, obesity, has been correlated with increased SAA values, while weight loss was associated with a decrease in SAA levels [123].

Increased values of SAA over 160 ug/mL in the first 12 h have been associated with the confirmation of atherothrombotic stroke [118].

In arteriopathic children with an arterial ischemic stroke, there was a correlation between increased SAA values and both occurrence and recurrence of stroke [124].

As a prognostic biomarker, SAA can be used as a predictor for patients at risk of developing a stroke-associated infection. Thus, by measuring SAA at admission, there is the possibility of identifying this particular patient group as at risk of having infectious complications, thus increasing mortality rates [125,126].

**Microalbuminuria (MAU)** is defined as the ratio between albumin and creatinine in urine. It is a proven biomarker for endothelial dysfunction in both kidney disease and cardiovascular disease [127].

One hypothesis regarding MAU states that it increases vascular permeability leading to lipid infiltration into the arterial wall, and thus has an active role in developing atherosclerotic plaques [128].

The relationship between MAU and common carotid artery intima-media thickness in non-insulin-dependent diabetes mellitus patients is well established. However, the exact correlation was discovered in non-diabetic patients, showing that MAU is directly linked to the atherosclerotic process [129].

Another study confirmed this, as the presence of MAU was associated with subclinical atherosclerosis in elderly patients with normal kidney function [130]. In addition, MAU is a predictor biomarker for the existence of internal carotid artery stenosis and represents a risk factor for event recurrence in TIA and minor strokes [128].

The incidence of MAU at admission in stroke patients was reported to be almost one-third of the total number of individuals, and it was correlated with early stroke severity and poor long-term prognosis, especially three-month clinical outcomes [127].

The poor short-term prognosis associated with the presence of MAU was confirmed by other studies, as well [131].

Nevertheless, in patients that survived a stroke and underwent neurorehabilitation, MAU represents a predictive sign for an increased rate of recurrent events, proving its valuable role as a prognostic biomarker [132].

Although no major studies have been performed on stroke patients that chose MAU as a target for therapy, several studies on hypertensive, diabetic, and patients with increased cardiovascular risk showed that MAU levels could be decreased by administering angiotensin receptor blockers or angiotensin-converting enzyme inhibitor [133,134].

**Pregnancy-associated plasma protein-A (PAPP-A)** is a protease from the metalloproteinase superfamily that breaks down insulin-like growth factor binding proteins, facilitating the action of insulin-like growth factors (IGFs) [135].

PAPP-A modulates the proliferation effect of the IGFs in the arterial wall, increasing its role in the atherosclerosis process. Even more, hyperexpression of PAPP-A is present, especially in unstable and ruptured plaques compared to stable ones [136].

Despite its role as a prenatal screening test for Down Syndrome, PAPP-A has shown potential value as a biomarker for cardiovascular and cerebrovascular disease, especially for the atherosclerosis process [137]. Moreover, in individuals with a high risk of cardiovascular events, such as diabetic hemodialysis patients, PAPP-A is associated with an increased risk of stroke and infectious complications [138].

High blood values of PAPP-A have been correlated with hypercholesterolemia and with the presence of carotid atherosclerosis [139].

Furthermore, PAPP-A is associated with unstable atherosclerotic plaques, both in asymptomatic and symptomatic patients [140,141].

PAPP-A levels increase in patients who have developed an ischemic stroke and have coronary artery disease [136].

In stroke patients not treated with heparin, a strong positive correlation was found between PAPP-A and stroke severity, quantified using NIHSS score. Still, in the same study, PAPP-A was identified as an independent predictor for poor outcomes [142].

The potential to decrease PAPP-A levels was observed in statin-treated patients compared to the control group [137].

**Matrix metalloproteinases (MMPs)** belong to the family of endopeptidases and have a distinguishable characteristic given by their dependence on metal ions, such as zinc. They have a significant role in the degradation of collagen, extracellular matrix, neurogenesis and regeneration, and myelin production [143]. From this family, two specific variants, **MMP-2** and **MMP-9**, emerge as having a direct link to ischemic stroke [144].

In vitro studies showed that MMPs play a distinct role in the plaque formation process and plaque instability [145].

Evidence of this was confirmed in vivo-studies that showed that MMP-2 and MMP-9 levels, intima-media thickness, and the stability of the plaques are strongly correlated with the ischemic stroke process [146].

The strong relationship between MMP-2, MMP-9, and plaques that were unstable and at risk of rupture, was confirmed by a separate study, as well [147].

Genetic polymorphism plays a specific role in the association between MMPs and ischemic stroke. Therefore, a couple of studies focused on this individual variability.

One study showed that the MMP-2-735C allele and the MMP-9-1562T allele are independent risk factors that increase the risk of ischemic stroke 1.5 fold compared to other genotypes [148]. In addition, the MMP-9-1562T genotype has also been proven in studies of both type 2 diabetics and non-diabetics to have a significant association with increased ischemic stroke risk [144].

Another study showed that the MMP-9 rs3918242 variant has an essential interaction with smoking and increases the risk of ischemic stroke [149].

DNA methylation is also a co-factor that can increase the risk of stroke, as lower levels of MMP-2 methylation have been found in ischemic stroke patients compared to the control group, especially in males [150].

High MMP-9 values in the early phase of stroke have been associated with poor disability outcomes and increased mortality risk [151].

The role of MMPs in the treatment of stroke patients has been a subject of discussion mostly because of their double role, both in the progression of injury in the acute phase and the neuroregeneration in the chronic phase of the stroke.

An experimental study on rats with MCA occlusion studied the possibility of extending the rt-PA treatment window up to 6 h from the onset by blocking MMPs activity with a specific inhibitor, minocycline. The results were promising and showed decreased MMP-9 values, reduced infarct volumes, and reduced rate of hemorrhage after rt-PA [152,153].

Statins represent another drug class that affects MMPs, by inhibiting their activity in the acute phase of ischemic stroke [154].

In an in silico study of a potential MMP-2 and MMP-9 inhibitor, phytochemicals derived from Withaniasomnifera interacted with the zinc ion found in the MMPs structure. These phytochemicals have excellent BBB penetration capability and could be a feasible drug that offers protection against neurological dysfunctions mediated by MMPs, such as ischemic stroke [155].

**Endostatin** is an endogenous angiogenesis inhibitor derived from collagen XVIII, which is part of the extracellular matrix in the endothelial and epithelial membranes [156].

Endostatin can suppress the proliferation and migration of endothelial cells, thus reducing the process of neovascularization, and has a direct role in the development of atherosclerotic plaque [136].

In a study performed on the Japanese population, it was observed that high endostatin values are independently correlated with subclinical atherosclerosis changes, such as common carotid artery intima-media thickness [157]. Furthermore, another analysis from the same cohort showed an association between carotid IMT and eGFR, while endostatin is more of a mediator in this relationship [156].

Another study found that increased endostatin values in patients with acute ischemic stroke are associated with severe neurological deficits and increased mortality risk, proving the role of endostatin as a prognostic biomarker [158].

The prognostic value of endostatin was further confirmed by other studies that showed that increased endostatin levels are associated with three-month cognitive impairment after an ischemic stroke [159].

**Vascular endothelial growth factor (VEGF)** is a protein produced by fibroblast cells and is acknowledged for its angiogenesis role. VEGF consists of five different members with similar characteristics.

VEGF is the counterpart of endostatin and has inverse effects. VEGF significantly impacts angiogenesis, especially over endothelial cell proliferation, by recruiting endothelial progenitor cells [160].

VEGF synthesis is regulated by various factors, such as the hypoxia-inducible factor, which appears in oxygen-deprived dysfunctions such as ischemic stroke [161,162].

A couple of studies proved the link between VEGF and arteriosclerosis. One study focused on the association between low VEGF plasma values and symptomatic intracranial arterial stenosis without association with extracranial arterial stenosis. In addition to that, the severity of the intracranial stenosis was directly correlated with the VEGF [161].

The pro-angiogenic activity of VEGF165, which is formed by splicing the VEGF-A variant, can further induce the development and progression of atherosclerosis [163].

In an experimental study on mice with Apoe-/- with pre-existent advanced atherosclerotic lesions, plaque instability was lowered by administering the VEGF-C variant [164].

In patients with atherosclerotic plaques, the production of VEGF could be isolated in both intraplaque polymorphonuclear neutrophils, as well as circulating ones [165].

Folic acid supplementation affects VEGF activity by inhibiting VEGF expression through DNA methylation and can slow down the atherosclerotic process by doing so [166].

Increased VEGF values in atherothrombotic stroke patients were observed without a significant correlation with carotid IMT value in the post-acute phase [167].

In the acute phase of the ischemic stroke, the VEGF levels were directly correlated with infarction volume, and higher values were observed in the large-vessel disease etiology compared to the small-vessel disease. In the same study, patients with increased VEGF values in the acute phase had a better three-month prognosis, with an improved NIHSS score [163].

In acute ischemic stroke, VEGF and endostatin values increased, with their peaks at day three and day five, respectively. Thus, a complex relationship exists between endostatin, VEGF, and circulating endothelial progenitor cells. In addition, the VEGF/endostatin ratio at day one and day three significantly correlated with the number of EPCs, a predictor for a good clinical prognosis [160].

**Limitations**

This review has a couple of limitations and shortcomings. The main limitation derives from the search method. Many articles from the literature could remain unidentified, either because search engine algorithms usually try to provide relevant data instead of exact data or because the search terms we decided to use could misidentify studies.

Another significant shortcoming of this review is that we did not classify or interpret any analytical data, such as biomarkers’ availability or cost.

**Summary of Evidence**

Each biomarker has different characteristics such as specificity and sensitivity (Table 2), interactions, and different data regarding the risk of developing stroke, the activity in the acute phase of atherothrombotic stroke, the outcome, and the interaction with the stroke treatment (Table 3).

## 4. Conclusions

These biomarkers offer new data that could improve clinical practice, but they cannot yet compete with the neuroimaging markers provided by computer tomography, magnetic resonance imaging, or Doppler ultrasound to detect or diagnose atherothrombotic stroke.

The current guidelines do not offer adequate data regarding the use of biomarkers in medical practice in either diagnosis or treatment. We suspect that this is because of already available advanced diagnostic techniques and the fact that few studies exist that attest to the importance and the role these molecules hold in stroke pathogenesis.

We think that there are some potential candidates that could rise as role models in the future, especially the ones that can be used for risk stratification, particularly in patients who have not yet suffered a stroke or had a transient ischemic attack. Of these, it is worth mentioning PAPP-A and VEGF. These could provide new ways of monitoring high-risk patients and maybe decide personalized therapeutic plans.

As for the diagnostic power of the atherothrombotic stroke, only a few have real potential, such as haptoglobin and serum amyloid A. Both showed high specificity and sensitivity for diagnostic. However, because there is no clinical experience and limited medical use, we think using any of these is premature.

However, some biomarkers can be used as additional tools to give supplementary data that could pinpoint possible stroke etiologies and even suggest future complications, such as post-stroke depression.

Another valuable piece of data derived from the use of these biomarkers is the predictive capabilities for stroke patient outcomes, especially short-term outcomes that could also influence acute phase treatment, such as rt-PA thrombolysis.

Future studies should be performed on viable biomarkers candidates on large cohorts of patients to assess their specificity and sensitivity properly, as most of the articles provided use reasonably small groups of patients.

Although it is anticipated that biomarkers could be of extreme value in the future, as precision and personalized medicine grows in popularity, the current level of evidence suggests that most of them are not recommended yet for regular clinical use, even if they can provide essential knowledge in selected cases.

## Figures and Tables

**Table 1:** Universal characteristics of biomarkers.

Universal Characteristics of Biomarkers
Non-invasive
Easily measured
Inexpensive
Produce rapid results
Measured from easily available sources (blood/urine)
Allow early detection
High sensitivity and specificity
Biomarker levels should vary according to therapy
Biomarker levels should aid in risk stratification
Biomarker levels should possess prognostic value
Evidence of the underlying disease mechanism and the role of the biomarker should be available

**Table 2:** Summary of evidence—Biomarkers’ specificity and sensibility.

Biomarker	Sensitivity	Specificity	Cut-off Value	Source
CRP—predictive of severe ischemic stroke	80%	75%	10.25 mg/L	Shoaeb et al. [25]
CRP—predictive of poor outcome	75%	82%	10.25 mg/L	Shoaeb et al. [25]
TAT—stroke diagnosis	58.1%	87.8%	1.75 ng/mL	Naifang et al. [29]
TAT—complete recanalisation	53%	89%	24 ug/L	Fernandez-Cadenas et al. [30]
a2-PI	NA	NA	NA	NA
PAI-1	NA	NA	NA	NA
sP-selectin	NA	NA	NA	NA
E-selectin—predictive of poor outcome	88%	76%	29 ng/mL	Richard et al. [51]
Adiponectin—moderate to high stroke diagnosis	63.6%	62.4%	7.0 ug/mL	Wang et al. [56]
Adiponectin—predictive of poor outcomes	78.6%	72.9%	9.0 ug/mL	Wang et al. [56]
Leptin—predictive of depression development at 1 month after stroke	86%	84%	20.7 ng/mL	Jiménez et al. [60]
Leptin—predictive of major depression within the first month after stroke	100%	99%	85 ng/mL	Jiménez et al. [60]
Leptin—day 1 concentration predictive of poor 90 days outcome	43%	92%	3.14 ng/mL	Carbone et al. [61]
Leptin/Adiponectin— day 1 concentration predictive of poor 90 days outcome	88%	61%	1.16	Carbone et al. [61]
Resistin	NA	NA	NA	NA
D-Dimer—diagnosis of acute aortic disection in stroke patients	100%	86%	4.1 ug/mL	Ohara et al. [74]
D-Dimer—stroke diagnosis	60.2%	88.9%	0.38 mg/L	Naifang et al. [29]
Hcy—predictive for post-stroke depression	82.5%	63.3%	16.5 mmol/L	Yan et al. [85]
ADMA	NA	NA	NA	NA
Lp(a)	NA	NA	NA	NA
Lp-PLA2 activity—predictive of 30-day recurrent stroke/TIA	78%	66%	207 nmol/mL/min	Delgado et al. [110]
Lp-PLA2 activity—predictive of recurrent vascular event	72%	59%	153.36 nmol/mL/min	Massot et al. [111]
Hp—diagnosis of atherothrombotic stroke	95%	88%	1040 ug/mL	Brea et al. [118]
SAA—diagnosis of atherothrombotic stroke	91%	83%	160 ug/mL	Brea et al. [118]
Microalbuminuria	NA	NA	NA	NA
PAPP-A—marker for plaque stability	100%	62.5%	0.395 ug/mL	Heider et al. [140]
PAPP-A—marker for pressence of neurological symptoms	82.8%	35.3%	0.555 ug/mL	Heider et al. [140]
MMP-9—predictor for MCA infarction development	64%	88%	140 ng/mL	Montaner et al. [153]
MMP-9—predictor for hemorhagic transformation after tPA	85%	79%	140-191 ng/mL	Montaner et al. [153]
Endostatin—predictor for death or severe disability	49.5%	60.31%	83.78 ng/mL	Zhang et al. [158]
VEGF—marker for intracranial atherosclerotic stenosis	93.1%	36.7%	64.05 pg/mL	Fang et al. [161]

**Table 3:** Summary of evidence—List of biomarkers.

Biomarker	Has Role in Risk Stratification?	Has Role in Diagnosis/Detection?	Has Role in Outcome?	Has Therapeutic Role? Or Is It Affected by Stroke Treatment?
C-reactive protein (CRP)	YES, high values are associated with increased risk of stroke and TIA, as well as increased risk of recurrence.	UNCLEAR, although high values appear in acute phases of stroke.	YES, high values are associated with poor outcomes, both in the short and long term. In addition, high values are associated with increased mortality.	YES, statins have been observed to reduce hs-CRP levels.
Thrombin-antithrombin complex (TAT)	Not enough data is available.	UNCLEAR, although higher values have been found in acute ischemic stroke patients.	YES, higher values are associated with poor outcomes.	YES, lower values are associated with better rt-PA success rates.
Alpha2-plasmin inhibitor (a2-PI)	YES, high plasma values are associated with increased stroke recurrence risk.	YES, higher values have been detected in patients with atherothrombotic stroke.	UNCLEAR, although in experimental studies, there was a slight reduction of brain lesion volume by administering human plasmin in order to inhibit a2-PI effects.	YES, higher values are associated with lower rt-PA success rates.
Plasminogen activator inhibitor-1 (PAI-1)	YES, high values are linked to stroke risk and stroke recurrence risk.	UNCLEAR, although higher PAI-1 levels can be found in stroke patients.	UNCLEAR, even if no statistically significant difference was found regarding PAI-1 polymorphism, there is an increased risk of hemorrhagic transformation.	YES, PAI-1 5G/5G is associated with an increased risk of hemorrhage after rt-PA treatment.
Soluble P-selectin (sP-selectin)	UNCLEAR, although increased values are found in patients with aortic atherosclerosis.	YES, high values have been found in stroke patients, with a decrease in value after three months.	YES, high values were found in progressive ischemic stroke.	YES, patients that received statin treatment showed a decrease in blood values of sP-selectin.
E-selectin	YES, genetic polymorphism is associated with increased stroke risk. Alleles association increases the risk even more.	Not enough data is available.	YES, independent predictor of poor three-month outcome	NEED FURTHER EVIDENCE. Experimental studies on mice showed improved outcomes after administering anti-E-selectin monoclonal antibodies.
Adiponectin (APN)	YES, higher values are associated with a lower prevalence of carotid atherosclerotic plaques.	YES, low values can be found at admission in stroke patients compared to control patients.	UNCLEAR, patients with higher levels at admission were more likely to have a poor outcome and increased mortality risk. However, low levels were associated with increased five-year mortality rates.	UNCLEAR. Further studies regarding a viable agonist of APN could hold the answer.
Leptin	YES, higher leptin values were associated with increased stroke incidence. Leptin/Adiponectin ratio could offer additional predictive value for subclinical atherosclerotic changes.	YES, both ischemic and hemorrhagic stroke patients had higher leptin values compared to healthy ones.	YES, patients with stroke history that had higher leptin values had an increased risk of recurrence. A high Leptin/Adiponectin ratio is associated with good outcomes.	YES, leptin values can be decreased by healthy lifestyle changes, statins, and antidiabetic drugs.
Resistin	YES, higher values of resistin are a risk factor for atherothrombotic stroke. The risk is further increased when diabetes or hypertension are present. Specific genotypes such as -420(C>G) are an additional risk factor.	YES, higher values have been found in the early phase of stroke. Experimental studies on mice showed resistin peak values around 12 h from the onset.	YES, high values of resistin were associated with increased stroke severity, increased disability after stroke, and a higher five-year mortality rate.	UNCLEAR. Additional studies need to be performed to confirm the evidence from experimental studies, where resistin was able to decrease brain edema.
D-Dimer (DDs)	YES, increased DDs value is associated with increased risk of ischemic stroke.	UNCLEAR, although in specific etiological subtypes of stroke, like occult cancer or undetected atrial fibrillation, it was shown that higher values of DD could be found. Because of the poor specificity and sensitivity of DD in stroke, the role is still unclear.	YES, higher DD values have been associated with progressive stroke. Also, combined with other biomarkers, it can be used as a predictive factor for the poor outcome of stroke patients.	UNCLEAR, although patients that had poor outcomes after rt-PA treatment had higher values compared to those who had good outcomes post rt-PA. This is suggestive of a decrease in the success rate of rt-PA in high D-dimers level patients.
Homocysteine (Hcy)	YES, high levels are associated with increased stroke risk. Also, patients that already had a stroke and high plasma values have an increased risk of recurrence.	YES, higher values were detected in stroke patients.	YES, higher blood values were correlated with higher severity of stroke using NIHSS score.	YES, a decrease in stroke risk was shown in groups of patients treated with folic acid in order to lower the total Hcy levels. Further studies are required to show the benefits in the general population.
Asymmetric dimethylarginine (ADMA)	YES, several studies showed that ADMA could be linked with the atherosclerosis process and with increased stroke risk.	YES, higher blood values of ADMA can be found in the acute phase of the stroke.	YES, higher values of ADMA have been associated with poor outcomes at 90 days after stroke.	UNCLEAR, rt-PA seems to decrease ADMA levels. A decrease in ADMA value was also seen in patients who received folic acid and B12 vitamin supplements.
Lipoprotein(a) (Lp (a))	YES, Lp (a) is a risk factor for both atherosclerosis and stroke, especially in young patients. High values are also associated with an increased risk of recurrence after a first-time stroke.	YES, higher values were found in stroke patients compared to healthy subjects.	YES, high values of Lp (a) are associated with poor short-term outcomes.	UNCLEAR, but increased values of Lp (a) were documented in patients that received statin treatment.
Lipoprotein lipase (LPL)	YES, polymorphism of LPL is a direct influence on the risk of developing ischemic stroke. Genetic variants have different effects in different ethnic populations, ranging from increasing stroke risk to having a protective effect. There is suggestive data showing LPL proatherogenic effect.	Not enough data.	Not enough data.	Not enough data.
Lipoprotein-associated phospholipase A2 (Lp-PLA2)	YES, high values are strongly associated with large-vessel disease and risk of transient ischemic attack, and also with a high risk of stroke reoccurrence.	YES, high values were found in patients with TIA and in stroke patients with arterial stenosis, especially intracranial stenosis.	YES, high values are associated with increased one-year mortality rates.	Not enough data.
Haptoglobin (Hp)	YES, Hp2 allele and its Hp2-2 genotype are associated with carotid atherosclerosis leading to potential strokes and TIA.	YES, high levels of Hp in the first 12 h of onset are strongly associated with atherothrombotic stroke.	YES, Hp2 allele is associated with premature cardiovascular deaths in patients with first-time ischemic stroke.	UNCLEAR, however, in the Hp2-2 genotype, there was an observed decrease of cardiovascular events after administering Vitamin E.
Serum amyloid A (SAA)	YES, in arteriopathic children with atherothrombotic stroke, SAA correlated with both occurrence and recurrence of stroke.	YES, high values of SAA in the first 12 h were associated with atherothrombotic stroke.	YES, patients with increased SAA at admission are more likely to develop infections, thus increasing mortality.	Not enough data.
Microalbuminuria (MAU)	YES, patients with MAU have an increased incidence of large vessel disease and recurrence of stroke.	UNCLEAR; however, MAU was detected in one-third of stroke patients.	YES, patients with MAU have a worse short-term and long-term prognosis.	UNCLEAR for stroke patients, but angiotensin-converting enzyme inhibitors and angiotensin receptor blockers have been shown to reduce MAU in patients with cardiovascular comorbidities.
Pregnancy-associated plasma protein-A (PAPP-A)	YES, PAPP-A has been associated with atherosclerosis, especially unstable atherosclerotic plaques.	YES, high PAPP-A levels are found in stroke patients that have coronary artery disease.	YES, patients with increased levels of PAPP-A have increased stroke severity and are more likely to have a poor outcome.	UNCLEAR, although statin treatment can decrease PAPP-A levels.
Matrix metalloproteinases (MMPs)	YES, high MMPs values are correlated with the presence of unstable atherosclerotic plaques. Also, specific genotypes have proven to be independent risk factors for ischemic stroke.	YES, high values of MMPs can be found in the acute phase of stroke.	YES, increased values of MMPs at admission are associated with poor outcomes and increased mortality risk.	YES, inhibition of MMPs activity in the acute phase of the stroke could be a viable treatment strategy. Statins are a drug class capable of decreasing MMPs activity. Other specific inhibitors could be used in future studies, such as minocycline alongside rt-PA, as a possibility to increase the therapeutic window and also reduce its secondary effects, such as brain hemorrhage. Another specific inhibitor could be phytochemicals derived from Withaniasomnifera, which have the ability to inhibit specifically MMP-2 and MMP-9 and have a potential neuroprotective effect against stroke.
Endostatin	YES, high endostatin values are associated with subclinical atherosclerosis changes.	YES, increased endostatin values can be found in stroke patients.	YES, increased endostatin values are associated with severe disability, increased mortality risk, and three-month cognitive impairment.	Not enough data.
VEGF	YES, low values of VEGF are correlated with intracranial arterial stenosis.	YES, High values of VEGF have been found in the acute phase of atherothrombotic stroke but with no correlation with atherosclerotic changes, such as IMT.	YES, high values of VEGF have been correlated with good clinical outcomes. The endostatin/VEGF ratio is another predictor for good outcomes.	UNCLEAR, administering VEGF-C to mice showed increased plaque stability. Acid folic affects VEGF DNA methylation and slows down the atherosclerotic process.
